# Interstitial Granulomatous Dermatitis Associated With Ulcerative Colitis: A Rare Cutaneous Manifestation

**DOI:** 10.7759/cureus.113843

**Published:** 2026-08-03

**Authors:** Madeena Mahmood, Kumayl Bhalloo

**Affiliations:** 1 Internal Medicine, Mersey West Lancashire Hospitals, Liverpool, GBR; 2 Internal Medicine, Mersey and West Lancashire Teaching Hospitals, Liverpool, GBR

**Keywords:** associated with ibd, general dermatology, interstitial granulomatous dermatitis, sweets syndrome, ulcerative colitis (uc)

## Abstract

Interstitial granulomatous dermatitis (IGD) is a rare inflammatory dermatosis that has been associated with several autoimmune conditions, including inflammatory bowel disease. Although cutaneous manifestations are well-recognised in ulcerative colitis, IGD remains an uncommon and under-recognised extraintestinal manifestation that can mimic other inflammatory skin disorders, leading to diagnostic uncertainty.

This case discusses a 36-year-old woman with a background of ulcerative colitis who presented with erythematous lesions affecting both forearms. Due to the clinical appearance of the lesions and the patient's underlying inflammatory condition, Sweet's syndrome was suspected. However, after further investigations from the dermatology department, histopathological examination demonstrated features consistent with IGD. The patient was treated with topical corticosteroids, resulting in the resolution of the lesions without recurrence.

This case underscores the importance of considering IGD as a differential in patients with inflammatory bowel disease who present with atypical cutaneous lesions and emphasises the role of histopathological examination in establishing the diagnosis and guiding appropriate management.

## Introduction

Interstitial granulomatous dermatitis (IGD) is a rare inflammatory dermatosis distinguished histologically by interstitial histiocytic infiltration with the dermal degeneration of collagen. It presents with erythematous to violaceous nodules, papules or plaques, commonly affecting the proximal limbs and trunk [1]. Due to its variable morphology, IGD can mimic other neutrophilic and inflammatory dermatoses, leading to diagnostic uncertainty until histological analysis can be done. 

IGD has been described in the literature in association with inflammatory and systemic autoimmune conditions, including systemic lupus erythematosus, rheumatoid arthritis, and inflammatory bowel disease (IBD) [2]. In ulcerative colitis, cutaneous manifestations are well-recognised, especially pyoderma gangrenosum and erythema nodosum, whereas IGD is an uncommon and underreported association in this population. Whilst the exact pathogenesis is not fully understood, it is believed to involve immune complex deposition and dysregulated inflammatory pathways [3].

The diagnosis is further complicated by the clinical overlap between IGD and neutrophilic dermatoses such as Sweet’s syndrome, particularly in patients with active systemic inflammation such as in IBD [4].

Histopathological analysis therefore plays a key role in differentiating these conditions and guiding the most appropriate management of this syndrome, especially in patients with active systemic inflammation such as in IBD [4]. Histopathological analysis therefore plays a key role in differentiating these conditions and thus guiding the most appropriate management. 

In this report, we present a case of IGD in a 36-year-old woman with a background of ulcerative colitis, in whom tender erythematous nodules were initially attributed to Sweet’s syndrome. This case highlights the importance of clinicopathological correlation and awareness of IGD as a potential, albeit rare, cutaneous manifestation in patients with inflammatory bowel disease.

## Case presentation

A 36-year-old female presented to the Emergency Department (ED) with a 2-day history of coffee-ground vomiting. During further assessment, oedematous, erythematous nodules affecting both forearms and multiple oral ulcerations were noted. The patient had a past medical history of ulcerative colitis (UC), for which she had undergone a panproctocolectomy in 2024, and gastroparesis. She also reported a family history of autoimmunity, with a paternal aunt diagnosed with vitiligo.

Observations were unremarkable. Blood tests demonstrated normal urea and electrolytes (U&Es), full blood count (FBC), and liver function tests (LFTs), with the exception of a mildly elevated white cell count (WCC) of 12.4 × 10⁹/L (reference range: 3.9-11.1 × 10⁹/L), C-reactive protein (CRP) of 59 mg/L (reference range: 0-9 mg/L), and alkaline phosphatase (ALP) of 154 U/L (reference range: 30-130 U/L).

Given the patient's history of UC and the clinical appearance of the lesions, Sweet's syndrome was initially considered the most likely diagnosis. She was subsequently referred to the dermatology department for further investigation. Upon review in the dermatology clinic, there were multiple slightly resolving erythematous nodules with centrally eroded areas on both forearms, which remained highly suspicious for Sweet's syndrome (Figure 1). Two 4-mm punch biopsies were therefore performed, comprising one lesional specimen for haematoxylin and eosin (H&E) staining and one perilesional specimen for direct immunofluorescence (DIF).

**Figure 1:**
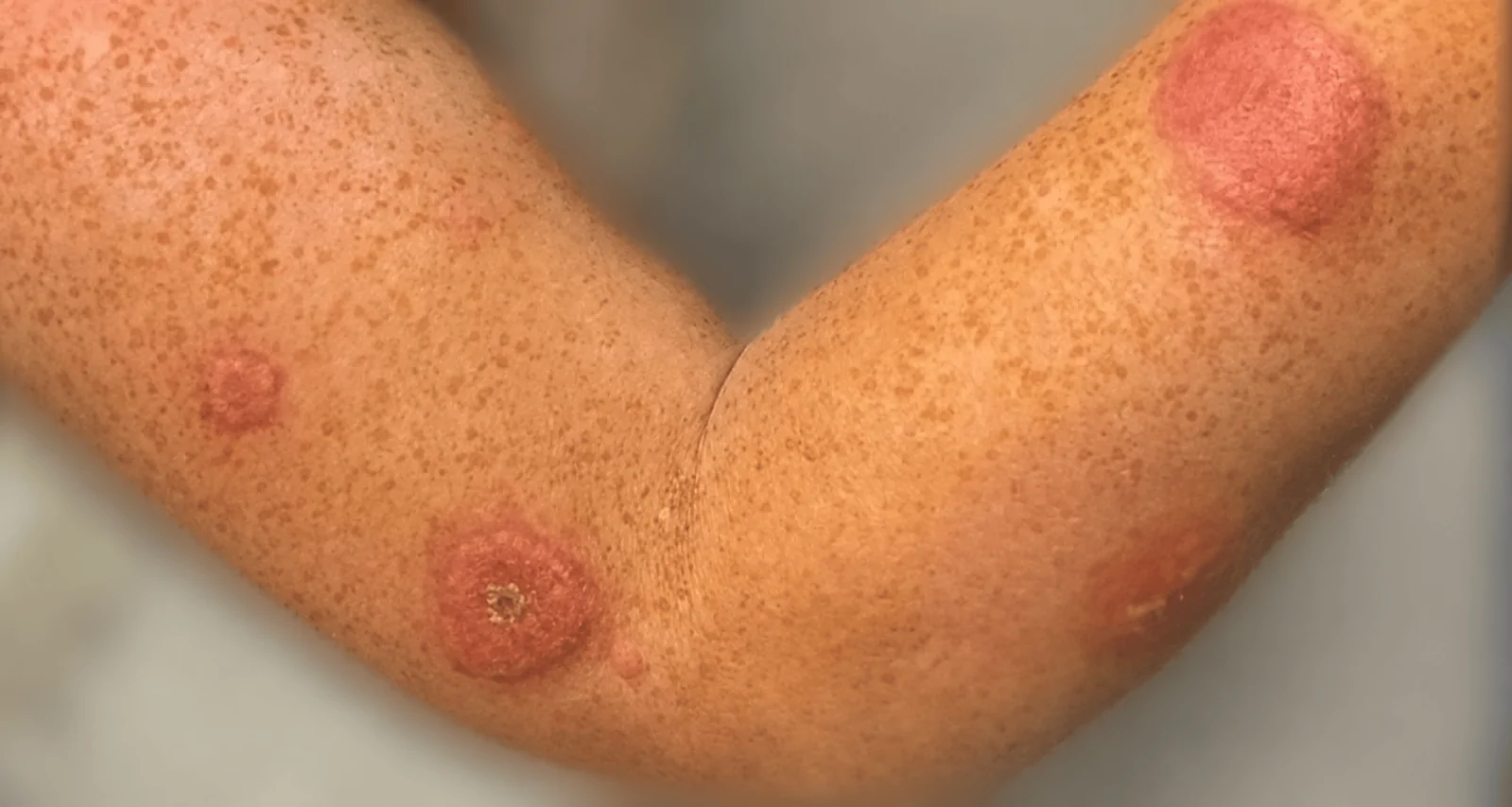
Clinical presentation of IGD showing multiple erythematous nodular lesions on the forearm IGD: interstitial granulomatous dermatitis

While awaiting histopathological results, the patient was treated empirically with topical corticosteroids for presumed Sweet's syndrome.

Histopathological examination demonstrated features consistent with interstitial granulomatous dermatitis (IGD), showing an interstitial infiltrate of histiocytes and lymphocytes with nuclear dust and red cell extravasation within the dermis. Eosinophils were present but relatively sparse, and intact neutrophils were absent. The overlying epidermis demonstrated mild spongiosis with lymphocytic exocytosis (Figures 2-4). DIF was negative for immunobullous disease. These findings established a diagnosis of IGD, representing a rare cutaneous manifestation associated with UC.

**Figure 2:**
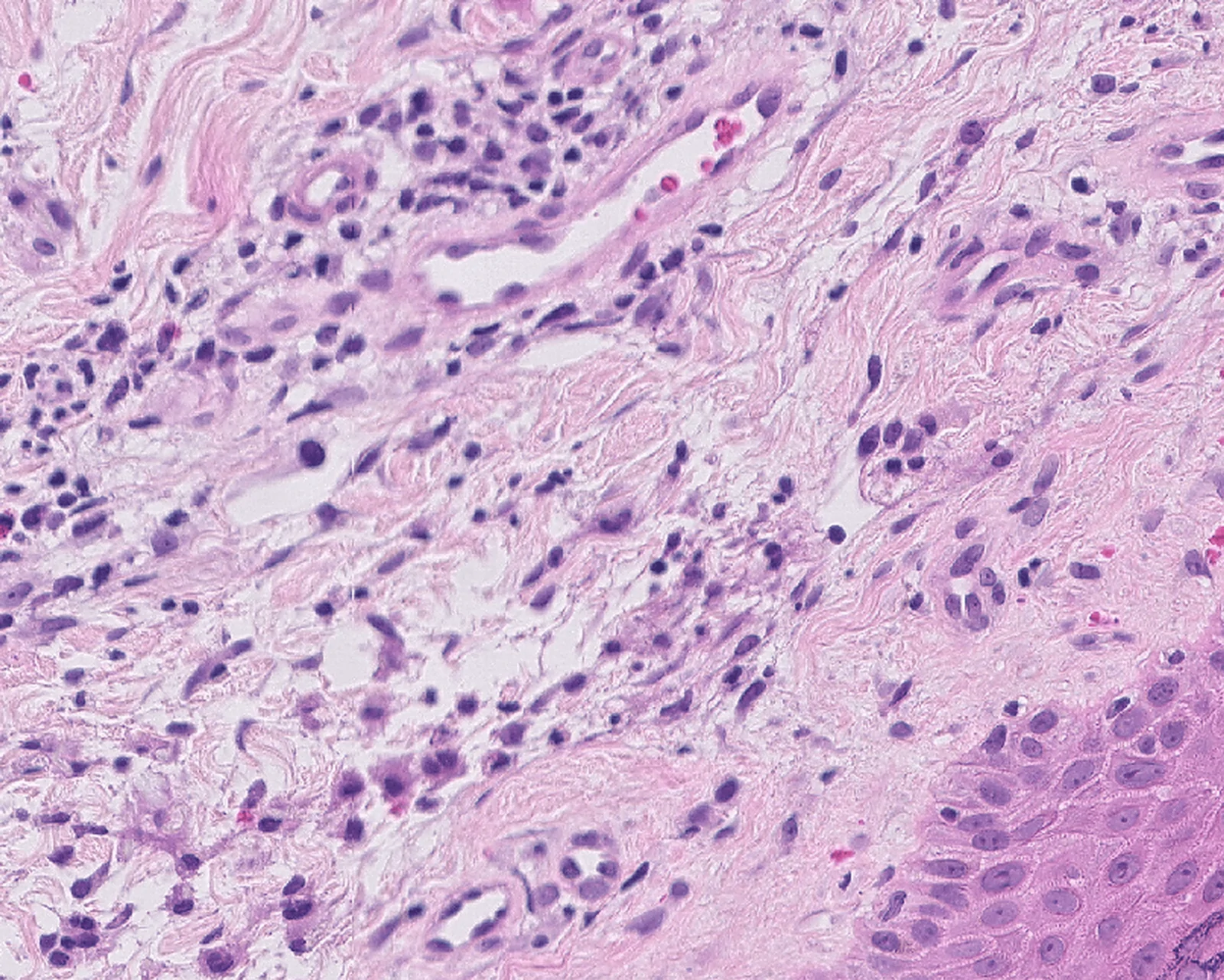
Histopathological features of IGD Haematoxylin and eosin-stained skin biopsy demonstrating histopathological features of interstitial granulomatous dermatitis, including an interstitial dermal infiltrate of histiocytes and lymphocytes with nuclear dust and red cell extravasation. Sparse eosinophils are present without intact neutrophils. The overlying epidermis shows mild spongiosis with lymphocytic exocytosis. IGD: interstitial granulomatous dermatitis

**Figure 3:**
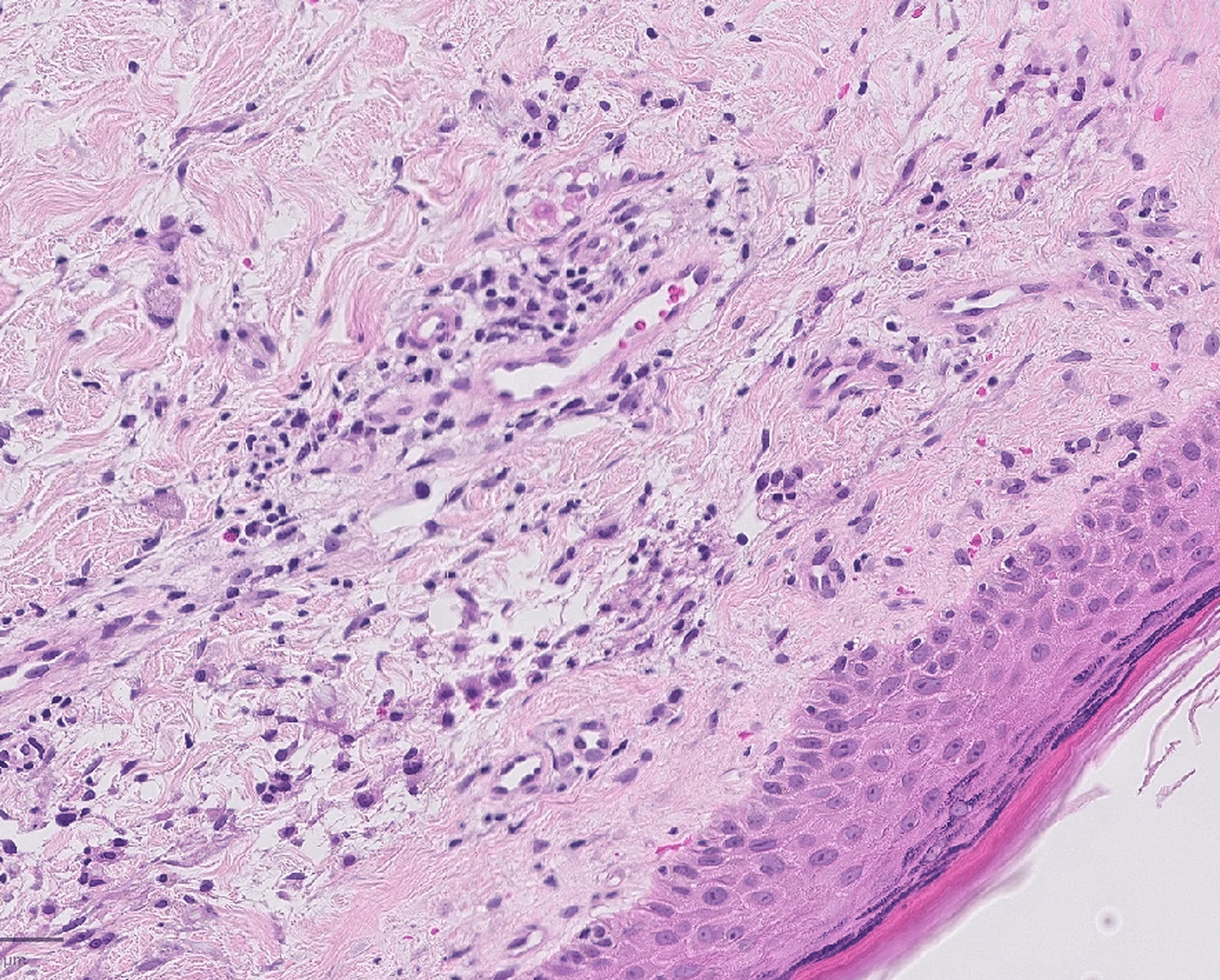
Histopathological features of IGD IGD: interstitial granulomatous dermatitis

**Figure 4:**
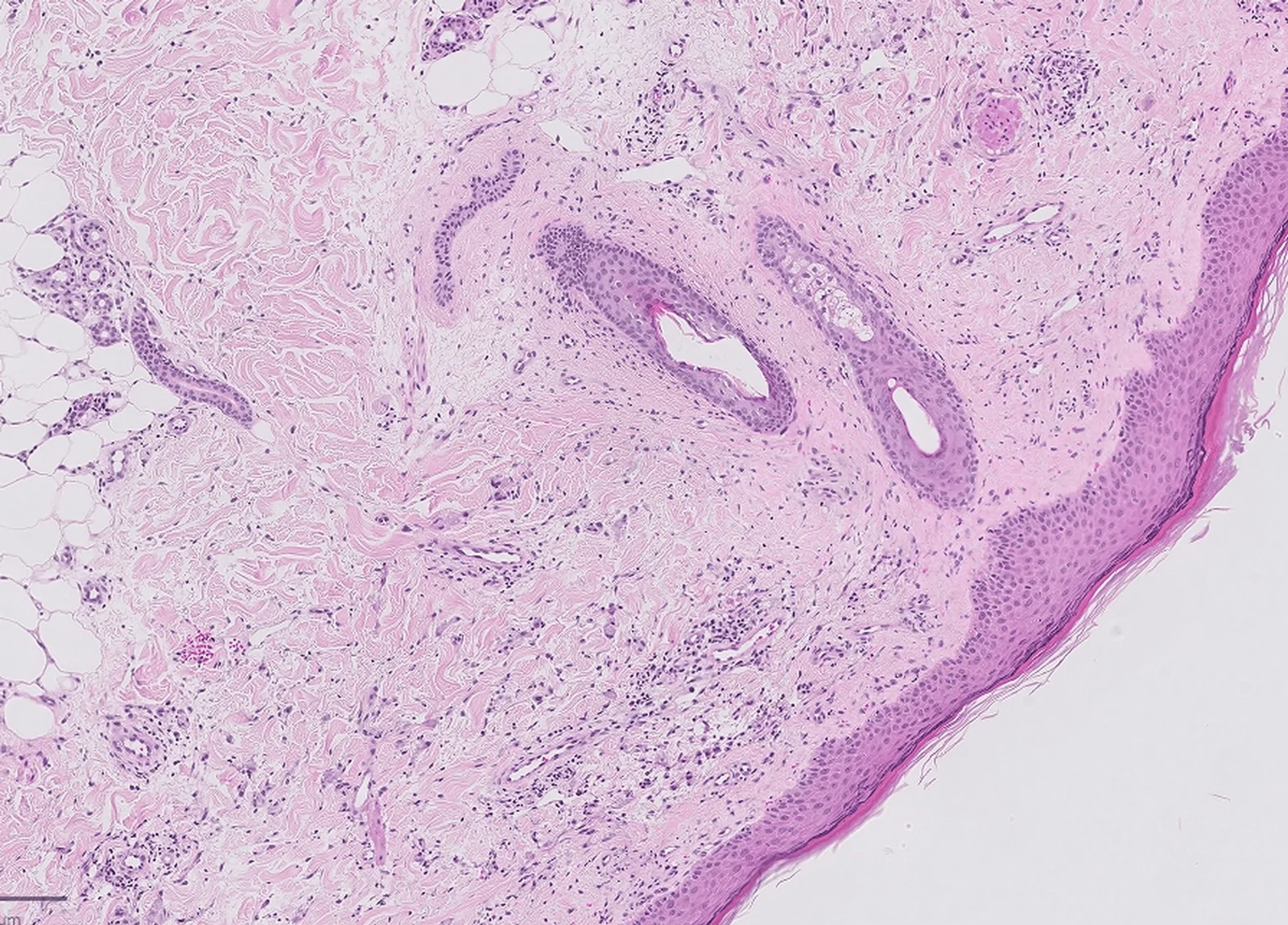
Histopathological features of IGD IGD: interstitial granulomatous dermatitis

The patient continued treatment with topical corticosteroids, resulting in resolution of the lesions. She was subsequently provided with an open follow-up appointment for six months should the lesions recur; however, she has not required any further review and remains symptom-free to date.

IGD is an uncommon inflammatory dermatosis that has been associated with several autoimmune conditions, most notably rheumatoid arthritis, systemic lupus erythematosus, and inflammatory bowel disease. Although cutaneous manifestations are well-recognised in UC, IGD remains a rare and likely under-recognised presentation. This case highlights the importance of considering alternative diagnoses in patients with inflammatory bowel disease presenting with atypical cutaneous lesions and demonstrates the crucial role of histopathological examination in establishing the correct diagnosis and guiding management. 

## Discussion

IGD is a rare inflammatory skin disorder that has been linked with several systemic autoimmune conditions, including systemic lupus erythematosus, rheumatoid arthritis and, less commonly, inflammatory bowel disease (IBD) [3,5]. Cutaneous manifestations affect up to 15% of patients with IBD and encompass a broad range of immune-mediated and reactive disorders, ranging from more common presentations like pyoderma gangrenosum, erythema nodosum and aphthous stomatitis to rarer granulomatous and neutrophilic dermatoses [6,7]. Careful and comprehensive clinical approaches are therefore required when evaluating these atypical presentations, as overlapping features can often complicate diagnosis [8]. In addition, the choice of IBD therapeutics like systemic corticosteroids, biologic agents, and immunomodulators plays a key role in guiding therapeutic approach, as these agents themselves can inadvertently trigger cutaneous reactions or can be used as mainstay treatments for both the intestinal and extraintestinal disease [7,8]. IGD, in contrast to other manifestations, is an uncommon and under-recognised extraintestinal finding, with a limited number of cases linked to ulcerative colitis. This rarity highlights the importance of clinicians recognising IGD as a key differential diagnosis when managing IBD patients who present with inflammatory skin lesions [3].

The pathogenesis of IGD is not fully understood, although it is thought to represent a reactive process linked with immune dysregulation. This is thought to contribute to degeneration of dermal collagen and the infiltration of histiocytes within the dermis, producing the characteristic histopathological appearance. In addition, the strong association between IGD and autoimmune conditions could suggest that systemic inflammation plays a role in disease development, although the exact pathway remains unclear [3].

An interesting feature of this case was the initial suspicion of Sweet’s syndrome. Both IGD and Sweet’s syndrome can present with tender erythematous plaques, nodules or papules in the context of systemic inflammatory disease, making clinical distinction challenging. Histopathological examination was therefore crucial to differentiate the two conditions, demonstrating an interstitial infiltrate of histiocytes and lymphocytes with degeneration of collagen, an absence of intact neutrophils and sparse eosinophils, features that are characteristic of IGD rather than Sweet’s syndrome, which typically shows a neutrophilic infiltrate without vasculitis [5]. This case highlights the important role of skin biopsy in establishing an accurate diagnosis when clinical findings can be non-specific.

Managing IGD mainly involves treating the underlying systemic inflammatory condition, alongside anti-inflammatory agents used directly on the skin. These reported treatments include topical or systemic corticosteroids, hydroxychloroquine, and steroid-sparing immunosuppressive therapies in refractory disease. Our patient responded well to topical corticosteroids alone with no recurrence of the disease in follow-up, suggesting that conservative management may be effective in patients with localised disease and well-controlled underlying ulcerative colitis [1].

## Conclusions

IGD is a rare skin manifestation of ulcerative colitis that can be difficult to recognise because it closely resembles other inflammatory dermatoses, including Sweet's syndrome. This case demonstrates the importance of considering IGD in patients with inflammatory bowel disease who present with atypical skin lesions. Histopathological examination was essential in confirming the diagnosis and directing treatment. Increased awareness of this uncommon association may facilitate earlier recognition, prevent misdiagnosis and contribute to a better understanding of extraintestinal manifestations associated with inflammatory bowel disease.
